# Social entrepreneurship in sport: a peripheral country perspective

**DOI:** 10.3389/fspor.2023.1256885

**Published:** 2023-10-25

**Authors:** Denise Kamyuka, Laura Misener, Marisa Tippett

**Affiliations:** School of Kinesiology, Western University, London, ON, Canada

**Keywords:** social entrepreneurship in sport, decolonial feminist research, integrative review, global south, peripheral country context.

## Abstract

For the past decade, scholars have been working towards developing a robust theory of social entrepreneurship in sport (SES). However, SES theory remains void of peripheral country perspectives and thus perpetuates the Eurocentric views of entrepreneurship. This paper used a decolonial feminist lens and Whittemore and Knafl’s methodology to conduct an integrated review of SES literature written in or about a peripheral country context. The review examined how scholarship from and about this context had considered geographical and culturally specific perspectives in the development of SES theory. A total of *n* = 1971 papers were retrieved, with only *n* = 12 providing relevant peripheral country context. This scarcity of literature indicates that the current theory of SES lacks peripheral country perspectives. Many papers in this review (*n* = 5) are written by authors in or from a peripheral country. Their contributions to SES literature revealed the decolonial feminist approaches that centralize alternative perspectives and added plurality to the definition of SES. The findings revealed the nuanced theoretical approaches to SES and highlighted the gaps in this context. The review shows how, despite the rise in social enterprises that focus on gender equity and the economic inclusion of women, gendered studies were still very scarce.

## 1. Introduction

For the past decade, scholars have been working towards developing a robust theory of social entrepreneurship in sport (SES) (1, 2). The frameworks used to conceptualize SES tend to be rooted in Eurocentric logic (i.e., the discourse that centers institutionalized Western European and North American ways of thinking.) and Eurocentric schools of thought on entrepreneurship (1). Consequently, much like sport for development, social entrepreneurship has been predominantly prescribed for economies in peripheral countries (3). It was, therefore, essential to examine how the development of SES theory—laden with colonial perspectives of entrepreneurship—considers peripheral country contexts through a decolonial lens.

Only literature on SES from a peripheral country context was considered in this integrated review. We reviewed the literature for alternative definitions and concepts in SES theory. We also examined how scholarship from and about this context had considered decolonial feminist (geographical, gendered, and culturally specific) perspectives in developing SES theory. ‘Peripheral country’ was coined by the Africanist Immanuel Wallerstein. He developed the World System Theory in rejection of Developmentalism theory and its essentialist, binary categorization of countries as developed or undeveloped. Wallerstein’s system acknowledged the power-grabbing tactics of the dominant core countries and considered the economic and social context that pushed some nations to the peripheries and pulled others to the core (4, 5). The term’ Global South’ is synonymous with ‘periphery country’ (the opposite of Global North/“core country”) and thus used interchangeably in this paper (6). Scholars have proposed that decolonial feminist approaches can help unpack alternative ways of understanding, particularly in conceptualizing entrepreneurial activities and theories in sports (7, 8). Thus, this peripheral country SES review used a feminist decolonizing perspective.

### 1.1. The option of a decolonial lens

Giraldo (9) offered the ‘option’ of a decolonial feminist lens, which invites scholars to challenge coloniality and the politics of knowledge that privilege English-language-dominated academia. A decolonial lens offers the option of broadening the conversation about colonialism to include discourse about modernity- the “visible side of coloniality” [(9), 160]. Coloniality refers to the power dynamics that long outlived colonialism (the act of colonizing) (10). qualified this notion of coloniality by describing a decolonial lens as a project that aims to make visible and undo the power dynamics of ‘epi-coloniality’ at work. Epi-coloniality describes the pervasiveness of colonialism that goes beyond systems to impact the power differentials in social hierarchies like race, class, and gender (also known as the ‘coloniality of gender.’ A decolonial project centers the voices of the colonized; makes them the primary audience; and prioritizes praxis, liberation, and justice as the outcomes of said project. In addition, Giraldo (9) emphasizes the insertion of a decolonial ‘feminist’ framework. They (ibid.) argue that this perspective was often forgotten in colonial theory and erased by modernity (which privileged Western European and North American feminism). A decolonial feminist sensibility invited a nuanced conversation that explores power dynamics across and at the intersections of race, gender, class, and culture. A decolonial feminist lens also prioritized praxis, solidarity, and community (11). According to (12) the difference between postcolonial feminism and decolonial feminism was that the latter addressed the influence of politics and governance on gendered lives, while the former assessed knowledge generated from allowing the voice and representation of females. This paper sought literature that accomplished the decolonial objectives.

## 2. Why social entrepreneurship in sport

Researchers have argued that African countries would benefit the most from social entrepreneurship, particularly in efforts to alleviate poverty, poor health systems, and education deficiencies (13). Social entrepreneurship is lauded for recognizing opportunities arising from the social deficiencies of a community and using local resources to fill these deficiencies (14–16), Social entrepreneurship is thus suitable for low to middle-income economies that are characterized by poor regulatory infrastructure, failing social sector institutions, large informal sectors, and economic instability (17–19) also suggested that social entrepreneurship was well-positioned to address gender inequality in the Global South. They (ibid.) posited that women facing economic precarity were inclined to engage in entrepreneurial activities for economic gains. Social entrepreneurship is therefore proposed for governments seeking to establish gender equality through inclusive economies (i.e., economies that tap into the unused economic potential of women). This rhetoric is adopted by sports for development (SFD) programs like the Nike Girl Effect Program, Women Win, and many more throughout the Global South [e.g., (20, 21)]. The prevalence of SFD programs for women was a testament to the growing sentiment that inclusive economies were essential for continued economic growth globally.

Like SES, SFD programs are criticized for perpetuating ideas of modernity (22). emphasizes the coloniality of gender in SES and SFD programs. In response, scholars have employed decolonial lenses that center the voices of the marginalized, although only a few scholars centered the voices of women and amplified their subjugated knowledges (23, 24). Applying this logic, this paper posits that without employing decolonial feminist perspectives, the definitions of SES will remain heavily entrenched in patriarchal and colonial ideologies. This paper argues that studies on SES from a Global South perspective would add plurality to the definitions and understandings of SES. By going a step further and adding a decolonial feminist lens, this review seeks to understand “Other(ed) perspectives” and theories from even the most marginalized voices [(25), 2]. These perspectives inform future research on SES in culturally relevant and contextual ways.

## 3. Concept definitions

### 3.1. Entrepreneurship vs. social entrepreneurship

Entrepreneurship is the act of creating a business with the goal of profit-making while assuming all the risk and uncertainty of the business failing (26). This definition of entrepreneurship varies from scholar to scholar. Therefore, it provides an amorphous foundation from which to define social entrepreneurship and (by extension) social entrepreneurship in sport. The main distinguishing factor between social and traditional entrepreneurship is the focus on a social mission (27). The most referenced definition of social entrepreneurship comes from (28). They (ibid.) describe social entrepreneurship as taking on a social mission, identifying opportunities to fulfill this social mission, creating value for the mission, and applying business principles to the innovative use of limited resources. Social missions begin by identifying a social problem. Social problems tend to be persistent issues requiring long-term and sustainable solutions (27). To ensure sustainability, social enterprises perform revenue-generating activities, whereby the profits are used to further the social mission and sustain the enterprise (29).

### 3.2. Social entrepreneurship in sport

As the name suggests, the only difference between social entrepreneurship in sport and social entrepreneurship (described above) is the addition of sport as the core industry, service, or product. The enterprise’s social mission is delivered directly through the sports activity (e.g., using a rugby program to keep out-of-school children off the streets) or as the main product/service, with sport being an auxiliary element of the programming (1). An example of the latter would be an enterprise like Alive and Kicking*,* which produces and sells soccer balls, but the organization also delivers free sexual reproduction education curricula to schools (30).

Previously, Dees reserved the definition of social entrepreneurship for non-profit organizations only. However, the profit potential of social enterprises has gained the attention of the for-profit business sector, resulting in the corporate adoption of social causes. The rise of the socially conscious consumer has incentivized private entities to apply cause marketing and adopt strategic social activities. Corporate social responsibility (CSR) is a corporate management strategy that allocates a budget to meet its stakeholders’ social or environmental interests (31). Therefore, CSR is exempt from the limited resources and bricolage characterizing traditional entrepreneurship. Unlike social entrepreneurship, CSR does not need to be guided by the values of the community; instead, it honors the desires of the shareholders (32). This distinction between CSR and social entrepreneurship was necessary for the decision to exclude CSR literature from this integrated review.

SFD refers to programs that use sport to achieve social and economic development outcomes, e.g., engaging at-risk youth; promoting peace and conflict resolution; sex and reproductive education; social entrepreneurship, and many more (33). Indeed, social entrepreneurship can be an alternative to as well as an outcome of SFD. Whereas SFD programs may get most of their funding from external donors, social enterprises primarily fund their operations by generating revenues as part of their entrepreneurial activities. This difference in funding models is why some scholars promote SES over SFD (34), as it provides a more sustainable option for development. SES was distinguished from SFD for this integrated review because it uses entrepreneurial activities for revenue generation, program sustainability, and fulfilling the social mission.

## 4. Methodology

### 4.1. Positionality statement: insider-outsider-within

I, the principal researcher in this integrated review, identify as a Black female academic from a former British colony in Africa. I am an insider (albeit in an essentialized manner) to the community of those who suffer from the sentence of colonial history (35). However, my position as an academic in North America complicates this connection to colonized communities. My academic qualifications are situated in Eurocentric logic and privilege Global North knowledge systems. The colonial system that governs my academic accomplishments creates a disconnect between me and the indigenous knowledge systems of other(ed) communities. I, therefore, identify as an ‘insider-outsider’ to communities in peripheral countries. The North American knowledge system presents those versed in it (i.e., conducting scientific research methods and presenting it as objective knowledge) as experts and authorities in their field of study. However, I found it hard to erase the decades of knowledge I had acquired from my community, elders, culture, and spirituality. This knowledge is too palpable and relevant to my lived experience for academia to ignore. Therefore, I also identify as an ‘Outsider-within’ the Global North knowledge system (36). More accurately, I identify as an Insider-Outsider-Within. The principal researcher is interested in identifying literature that amplifies alternative perspectives on SES that affirm those of my own other(ed) community. As a female, I am particularly interested in scholarly work that challenges coloniality in academia and gender by demonstrating critical reflexivity in combination with a decolonial feminist lens.

### 4.2. Integrative review method

We used Covidence software to upload database searches and perform an integrative review of SES literature from a peripheral country context. An integrative review methodology analyses theoretical and empirical research, thus allowing for a broad review of primary works of literature. This flexibility is essential where there is a dearth of literature on a topic. An integrative review is used for a multiplicity of purposes, like defining concepts and reviewing theories and methodologies. By including a multiplicity of sample frames, an integrative review increases the possibility of capturing “the complexity of varied perspectives” that could contribute to the comprehensive development of SES theory [(37), 663]. The integrative review followed the principles of (38) methodology. The critical considerations for this methodology involved developing a data analysis strategy that outlined what information was relevant and irrelevant for data extraction. We made sure to explicitly define the purpose of the research, the terminologies and concepts, and the steps for analysis.

We met several times before the data collection began. We discussed the existing phenomena relevant to the intersection of sport and social entrepreneurship. We concluded that critique on sport for development has increased (33, 39, 40). We found there was also an increase in scholarly discourse on the institutionalization of corporate social responsibility in sport (41, 42), sport philanthropy (43–45), and socially responsible athletes (46–52). These concepts often included a component of social entrepreneurship. This existing discourse was the rationale for the previous section that outlined the difference between entrepreneurship, CSR, SFD, and social entrepreneurship. This step narrowed the literature search and clarified the inclusion and exclusion criteria.

#### 4.2.1. Problem formulation

The challenge with integrated reviews is that they integrate a variety of variables, topics, samples, and demographics; this can make it hard to maintain rigor and stay on track with what is relevant and irrelevant information (38). recommend that integrative reviews should have a well-defined problem formulation process. The problem should articulate the purpose of the review. They (ibid.) advocate for more integrative reviews that apply philosophical, theoretical, and broad perspectives rather than just describing literature.

Ultimately, we developed the following problem statement: In response to calls to further refine the theory of social entrepreneurship in sport (1, 2, 53). The lack of articles from a peripheral country context in (1) review of social entrepreneurship in sport research confirms this gap in the literature. This paper reviews the available literature from a peripheral country context. We analyzed the literature for alternative perspectives on SES theory. To provide alternative perspectives to the Eurocentric discourse on SES, scholars posit an approach that intentionally confronts colonial legacies (22, 34). Therefore, we propose a decolonial feminist lens as the most thorough approach, as it includes the perspectives of the most marginalized (colonized) voices. In contrast to Bjärsholm’s review, this integrative review looks at the literature on SES strictly from a peripheral country context. We analyzed the authors’ theoretical and methodological considerations for these explicit contexts.

#### 4.2.2. Literature search

The following method for conducting the literature search is documented to confirm rigor in selecting a maximum number of suitable sources (38). We conducted a literature search in the following ten databases, in consultation with a research librarian (who specializes in reviews): Web of Science, Scopus, SportDiscus (EbscoHost), Academic Search Ultimate (EbscoHost), ABI Inform Global (Proquest), ABI Inform Dateline (Proquest), ABI Inform Trade & Industry (Proquest), Business Source Complete (EbscoHost), Sports Medicine & Education Index (Proquest), Sociology Collection (Proquest). The searches were conducted in December 2021 and updated again in December 2022 to identify additional literature. Appropriate subject headings and numerous synonyms for the following main concepts were strategically combined and searched: Concept (1) Social entrepreneurship*, Concept (2) Sport*, Concept (3) Peripheral countries (54).

Most peripheral countries were colonies of Western European colonial powers, such as Portugal, Spain, France, and England. For that reason, the search included literature in those four languages.

Phase one included a literature search with no filters or limits on dates and types of publication. A total of *n* = 2805 were de-duplicated using Covidence. *N* = 835 entries were deleted. The search terms included all possible synonyms for entrepreneurship* and a list of as many sports as possible. The titles and abstracts of the retrieved articles were added into Covidence.

In phase two, *n* = 1970 abstracts were reviewed by two reviewers, and a third reviewer resolved conflicts. Abstracts that had two yes votes moved onto the full-text phase. The abstracts were reviewed for the mention of social entrepreneurship* AND sport*. Due to the dearth of research on the topic and the varied definitions of social entrepreneurship in sports, articles mentioning “sports entrepreneurship” in the title or abstract were also included. At this stage, articles not written in a peripheral country context, articles that focused on sport for development with no mention of entrepreneurship, and articles that focused on corporate social responsibility were excluded.

In phase three, *n* = 137 articles were accepted, and the full texts were uploaded to Covidence for further review. Non- peer-reviewed articles were excluded. Newspaper articles were also excluded due to a lack of meaningful data on the theoretical and methodological approaches they used. Two reviewers reviewed each full-text article. A third reviewer resolved conflicts. Articles with two approvals were accepted and included in this review for data analysis. This process resulted in *n* = 12 articles being used for this review.

#### 4.2.3. Data evaluation and analysis

Only peer-reviewed articles were included in this literature search, and there was such a small sample reviewed in the end. As a result, it was not necessary to perform a quality appraisal of the literature (37).

A mixed-method and qualitative design of data analysis are cited from Miles and Huberman (55) as the most applicable designs for integrative reviews. Namely [(38), 550] recommend the following process: “data reduction, data display, data comparison, conclusion drawing and then verification.” First, the authors grouped the selected articles into quantitative and qualitative data. The data was reduced into subgroups that systematically compared definitions, methodologies, theoretical concepts, geographical context, and a gendered focus. This information is then displayed in an Excel spreadsheet. The articles were revisited several times to determine the common themes and concepts. These concepts were compared to the definitions and theories on SES previously detailed in this review. Any deviation or additions to these definitions or theories were added to the table. The theoretical and methodological approaches were interpreted for their use of decolonial feminist approaches.

Find the findings in Supplementary Table S1 (56). criteria were used to determine whether a concept, theory, or methodology satisfied the call for decolonial approaches.

The research has an element of:

1. Reflexivity.
2. Being borne in a local context.
3. Alternative onto-epistemological logic (Alternative to positivist, technoscientific logic).
4. Local language and colloquial preservation.
5. Practical application or implications for the local context (not just academia)

To meet the call for female voices (23, 57), the articles were also analyzed to focus on gender.

## 5. Results

We iteratively reviewed the data and our comparisons. We explored various patterns, themes, and conclusions. The conclusions were then shared and discussed with social entrepreneurs in sport from a peripheral country context and other scholars studying SES. Interpretations and conclusions were made for each subgroup. These conclusions are presented in Supplementary Table S2.

A total of *n* = 1971 papers were retrieved from the literature search. Only *n* = 12 papers discussed social entrepreneurship in sport from a peripheral or semi-peripheral context. Studies have explored social entrepreneurship’s cultural and geographical contexts see (58); however, similar scholarship in sport literature is gravely lacking. This lack of SES literature from a peripheral country context indicates that the theory of SES was developing, deprived of peripheral country perspectives.

All *n* = 12 studies espoused (28) definition of social entrepreneurship, describing it as innovatively fulfilling a social mission. Across all studies, scholars maintained Dee’s concept of social entrepreneurship. However, the organizational forms of SES varied. The bulk of the papers explored community enterprises (*n* = 2, 16%), which involved concepts such as community-based social enterprise, shared economy (59), and global strategic community relations programs (GSCR) (60).

Twenty-five percent (*n* = 3) of the articles used ’social innovation.’ Only three (*n* = 3) articles applied a decolonial feminist lens. Nearly half (*n* = 6) of the studies had a gendered component, briefly mentioning women in the study.

Only two papers used quantitative methods, namely questionnaires. The rest use qualitative methods (*n* = 10), like case studies (*n* = 3), semi-structured interviews (*n* = 5), and ethnographies (*n* = 2). The explicit use of decolonial methodologies was mentioned in *n* = 3 papers, namely, (40, 60, 61) work in Uganda.

East Africa was featured in *n* = 4 papers, making it the region most featured. Surprisingly, almost half the papers (*n* = 5) were written by authors from or living in the country being studied and encultured in the local context.

## 6. Discussion

### 6.1. Theories, concepts, and definitions of SES

In all three concepts of community, ‘community’ was prioritized over revenue generation. The community was responsible for developing, investing, and making the enterprise successful. The community determined whether the innovation had social value. The local entrepreneur built a rapport with the community and leveraged this connection to get them to buy into the solution. Therefore, the enterprise’s success depended on the community’s acceptance of and interest in the enterprise. Community enterprises are organizations that innovate products or services to meet the social needs of whole communities (62). Community enterprises “typically attempt to stimulate social and cultural life, increase business development, and strengthen community identity with the aim of building community resilience” (ibid. p. 2). However, this definition was used with caution, as it is derived from a Eurocentric context, where depopulation was often the impetus of social entrepreneurship (62).

Hayhurst (60) cited King (63) to describe GSCR as relationships between corporations’ social strategies and the community. Hayhurst’s (40, 60, 61) papers were not excluded because they centered on the community and the entrepreneurial desires and demands of the community over the corporations’ goals (22). paper on the “Girl Effect” amplified the experiences of the enterprise, the participants, and the community over that of transnational corporations. Admittedly, excluding other literature that mentioned CSR (during phase one) may have prevented the researchers from finding more articles that emphasized the community and community-run programs instead of the corporate’s interests. However, engaging in such exploration would have adjusted the scope of this review.

The term’ social innovation’ was included in the literature search as a synonym for social entrepreneurship. According to (64), social entrepreneurship—setting communal strategies and promoting social change—provides the mechanism for social innovation. Much like ‘community enterprises,’ the value of the innovation and, subsequently, the success of the innovation is determined by the invested interest of the community.

These articles on social innovation were selected for the review because they describe innovation as a tool for entrepreneurship that meets a social need (65, 66) introduced social inclusion in sport as a social innovation that creates value by bringing about social awareness and social understanding about marginalized populations. Social inclusion moves the economy forward by “serving a new market” (28, 67). This contribution broadens the scope of social entrepreneurship. Therefore, scholars can define a social enterprise as an enterprise that finds innovative ways to deepen the inclusion of historically marginalized populations (i.e., inclusion in decision-making, product/service design, and other operational or functional aspects of the organization). This connection between ‘innovation’ and ‘inclusion’ in sport is further validated by González-Serrano et al. (68) bibliometric analysis of sport literature. The analysis found that the keywords ’social innovation’ and ‘entrepreneurship in sports’ appeared as a prominent cluster in the literature (p 19). The concepts have developed into a distinct field of study in sport. Within this field, the concept of ’Sports Innovation for Inclusion’ was sighted the most.

There are two examples (*n* = 2) of literature borne in a local context that linked social innovation (in the form of social inclusion) to entrepreneurship in sport. First (66), use context-specific literature to develop meanings for social innovation. They applied a case study of a local Brazilian athlete from a marginalized community who became a social innovator by targeting people from similar backgrounds as beneficiaries of his social enterprise. Secondly (61, 69), explain how the social inclusion of women can bring about social change and economic growth. They explored contexts where locals pursued economic independence by challenging the gendered use and perceptions of bicycles (61). had an encultured researcher, the team, who used their familiarity with the context to assign meaning to the local perceptions about bicycles. The encultured researcher also provided a colloquial context to how positive deviance from the gendered use of bicycles fostered inclusivity for women and girls. These examples, from a Global South perspective, help to develop more comprehensive concepts in sport scholarship that encompass a plurality of lived experiences and cultures. In this case, social inclusion was centered as an approach for SES.

Feminist researchers in management studies have called for research that explores nuanced and gendered ways of creating social value (i.e., enterprising) (57). Ljunggren and Sundin (70) invited scholars to look for innovation that stems from the microphenomena experienced by marginalized communities, mainly how value was created and quantified outside of capitalist economic imperatives (23). encourage scholars to view decolonial feminism as a critical lens for sense-making rather than a theory or methodology. In doing so, scholars were invited to look at gendered power relations as more than just female oppression and male power. Instead, a feminist lens requests that researchers critically assess whose voices were being centered and whose were being pushed to the peripheries. Hayhurst’s work was the only work in this selection of articles (*n* = 3) that explicitly applies this decolonial feminist lens to center alternative perspectives.

The work on bike-sharing and bicycle for development (BFD) of (61) features prominently in this review (*n* = 3). This work straddles the lines of entrepreneurship and sport for development but also offers some of the only works that engage in feminist decolonial perspectives of SES.

### 6.2. Gender focus

These studies (*n* = 6) did not provide enough detail on the nuances of gender in a peripheral country context, nor did they assess gendered leadership in SES. Only three studies—all authored by (22, 60, 71)—centered the female perspective by introducing the female voice using decolonial-feminist methodologies. Despite including the female perspective, Hayhurst (ibid) only explored women participating in SES programs; no studies focused on SES that were women-led. Her research introduced interpretations of the word ‘empowerment’ and the concept of ‘entrepreneur as a sole hero’ that questioned the dominant language used to describe social entrepreneurship (34, 40). Without the voices and perspectives of women from the Global South, the masculine and Eurocentric dominance in entrepreneurship and the fallacy of universal feminism (11, 40) remains unchallenged and threatens to perpetuate academia’s erasure and oppression of women in the Global South. Female voices of the ’subaltern’ often challenge the discourse of modernity (72).

New interpretations, based on peripheral country logic, could also challenge the entrepreneurial criterion and characteristics used to identify enterprises and entrepreneurs (respectively). By homing in on the gendered component, This research added to the call for gendered perspectives in sport scholarship and knowledge production (22, 57, 67, 73).

### 6.3. Methodological approaches

Authors of studies in a peripheral country context used various qualitative methods of inquiry to explore the concept of SES. The two papers that employed a quantitative questionnaire were from a semi-peripheral country context, specifically a Croatian or Portuguese context. These studies used a broad-based/universal understanding of social entrepreneurship; they quantified the phenomenon rather than exploring the concepts in SES. An objective of this paper was to determine whether alternative perspectives were included in the development of the concept of SES. We argued that critical decolonial feminist thought provided an exploratory framework that grounded theory and concepts in alternative perspectives. To determine whether a decolonial feminist lens was applied, the literature was analyzed for the use of critical approaches. The section below discusses five critical approaches of decolonial feminist work and how the articles employed them.

#### 6.3.1. Reflexivity

Reflexivity was not apparent in many of the studies (*n* = 7) and thus did not overtly acknowledge the geopolitics of neoliberalism, i.e., the Global North and Global South power divide. In the studies that did use reflexivity, decolonial practices extended beyond the researcher’s reflections on their privilege, power, and bias (74). Ultimately, critical reflexivity ensured that all five forethoughts for decolonization were incorporated into the research design and applied at all stages of the research (25, 61, 75) conducted their methodology through a critical interpretivist lens. Throughout the study, they reflected on how individuals and organizations made sense of and derived meaning from social phenomena. Under a critical interpretivist lens, the authors assumed that society forms meaning and interpretations based on local circumstances and contexts. This lens employs reflexivity and takes care not to alter the local meaning of things for the purposes of academic consumption. By preserving the local meaning of things, the authors acknowledged epistemological value in local definitions and meanings (25) posited that any research that exercises reflexivity to challenge colonial legacies, assumptions about epistemologies, and power dynamics is engaging in some degree of decolonization.

#### 6.3.2. Borne from the local context

The cultural, historical, and material contexts were firmly attached to sense-making and social reality. In their study (61), contextualized political and social considerations with an overview of Uganda’s history. The research and the solutions from the research addressed the issues that the local community and elders saw as a priority. The research design and questions were informed by local stakeholders and customized to the local context. Understanding the local context helped to identify the onto-epistemological roots interpretation. “Having an encultured informant who is from and lives in Uganda as a crucial member of the research team from the inception of the project until the writing of the project was crucial in sensitizing members of the team not familiar with Uganda to the range of local issues and power dynamics issues in and around research locations” (p. 14).

#### 6.3.3. Alternative onto-epistemological logic (alternative to positivist, technoscientific logic)

There is a lack of research that seeks to understand humans as social beings and how relational knowledge impacts the definitions of social entrepreneurship (56, 76) described ontologies from the Global South as relational. The most important relationship being that of ontological-epistemological re-existence, i.e., recognizing alternative sites of knowledge (e.g., the spiritual realm, the body, the land) and connecting these sites to rigorous ways of knowing (72). Despite indigenous research and decolonial research scholars’ calling for the acknowledgment of alternative ways of knowing, none of the papers selected in this review cultivated knowledge from alternative sites of onto-epistemology.

#### 6.3.4. Local language and colloquial preservation

Hayhurst’s (40) research used the word ‘empowerment’ to demonstrate how meaning could be lost or misinterpreted when words are translated from a Global North to a local discourse and vice versa. She contextualized and preserved the local meaning of the word Ndlovu-Gatshei (77). In their findings (61), discovered that the word bicycle held different meanings depending on the context. “Respondents told us about the bicycle as a shared ‘village bicycle,’ a tool for the poor, a signifier of illness (HIV/AIDS), and more” (p. 40). In centering the local voice and meanings, these papers open a window into an alternative interpretation and practice of SES. They invite scholars to further explore and glean from the knowledge held in these alternative meanings.

The rest of the papers had no explicit consideration for linguistics. This omission of local languages threatens to reinforce the power dynamics that govern the politics of knowledge production (56). It makes a statement about who the research is for, what languages knowledge can be held in, and the need for foreign interventions to produce knowledge (78).

#### 6.3.5. Practical application for local context

The literature did not demonstrate practical implications that go beyond academia and explore the local and practical application of the research (10, 25, 79). According to Smith (78), the knowledge from research is disseminated back to the locals it was mined from, in languages and theories that make this knowledge unrecognizable to the locals. This ‘newly produced’ knowledge is then prescribed for the local context and administered by ‘experts’ from the Global North (61) conducted research that engaged the local community from the beginning of the research. They focused on the issues the community wanted to focus on and the solutions it wanted to see implemented. The community also decided how they wanted the information to be disseminated. The organizations in the (61) paper did not want to be anonymous in the dissemination of the research. Practically, this served as a means of marketing the work of BFD organizations in Uganda and served as an example of success. Their (ibid.) conclusion also provided practical reflections that could improve the rollout of BFD projects in other communities.

### 6.4. Geographical context

“Where the research grows from and who funds it matters as much as if not more than the kinds of research methods/strategies used or the theoretical frameworks that inform such work” (72, 80). This quote captures the principle of geographical context within decolonial feminist work. It recognizes that geopolitical lines that favor the Global North are embedded in the politics of knowledge. The encultured scholars did not consider an overt decolonial lens that aimed to de-center Eurocentric knowledge.

Encultured researchers may not feel equipped to conduct decolonial research. Perhaps decolonization was not at the forefront of their academic agenda. Perhaps they do not grapple with racial and neocolonial power dynamics like their White counterparts and thus exercise less reflexivity. Perhaps at this stage, getting work from a peripheral perspective published was the first and most significant battle; the luxury of taking seemingly less rigorous approaches to research may be too much of a risk for scholars who need publication to advance their careers.

## 7. Conclusion and future considerations

Authors (particularly scholars from the dominant North) often enter new geographical contexts seeking something novel to share with the academic world. This need for knowledge production often stripped communities of their indigenous knowledge and repackaged it in jargon, theory, and other academic devices (78). This capitalist approach left the purpose of research and the dissemination of research up to the researcher instead of allowing the community to decide on the issues they wanted to focus on and the solutions they wanted to see. The Canadian Research Ethics Board has increased ethical awareness on how research should engage the community it was researching (with). Practical dissemination and application of research to and for the community has become a requirement for most studies. However, these requirements were amiss in not requiring that researchers commit to celebrating and re-affirming a community’s indigenous ways of being, knowing, and doing (72). Without institutional acknowledgment of indigenous knowledge, European and North American academia will continue to dismiss a plurality of knowledge in preference of ‘othering,’ demeaning, and marginalizing alternative ways of knowing.

Coupled with the need to celebrate indigenous knowledge is the need for epistemic justice and liberation (77). Creating opportunities and spaces for indigenous researchers to grow in epistemic freedom, get trained in decolonial methodologies, and become versed in decolonial approaches is integral to indigenous knowledge cultivation. Contemporary research methodologies embody decolonial tenets, like guided collaborative autoethnographies (81–83), counter-storytelling (84), and feminist participatory action research, can be used to democratize research and center alternative perspectives in sport scholarship.

It is encouraging to see authors in peripheral countries researching social entrepreneurship. It is also exciting to see authors apply elements of decolonial approaches to their research (whether consciously or subconsciously). One may hypothesize that as more sport scholars from the Global South (much like me) find their place in the world of academia, more assertions of decolonial tenets will appear in their scholarship. The struggle with decolonial work for Global South scholars is the need to first ‘decolonize the self’ and critically apply, reject, or manipulate dominant Global North discourse (10, 72, 85). However, a call to intentionally apply decolonial feminist critical sensibilities to decolonizing research cannot be ignored or put off. Due to the pervasiveness of patriarchy in ’sport’ and ‘entrepreneurship,’ this call cannot be taken passively; decolonial feminist approaches require intentionality. Gathering alternative perspectives from this decolonial lens will further add plurality to the definitions of key concepts in sport literature. This approach dispels the fallacy of a universal definition (which serves the dominant discourse of Global North academia), favoring a multiplicity of small-batch and nuanced definitions with practical applications for specific communities.

## Data Availability

The original contributions presented in the study are included in the article/**Supplementary Material**, further inquiries can be directed to the corresponding author.
