# Supplementary material for: Social entrepreneurship in sport: a peripheral country perspective
Source: Front Sports Act Living. 2023 Oct 25;5:1256885. doi: 10.3389/fspor.2023.1256885 (PMC10642194; doi:10.3389/fspor.2023.1256885)
Supplement: Supplementary file 1 [file Datasheet1.docx]

Supplementary Material

Social entrepreneurship in sport: a peripheral country perspective

# Table of data

| **Article** | **Geographical context** | **Methodological approach** | **Theoretical approach, Concepts and Definitions** | **Gendered** | **Decolonial component falcón, (2016)** |
| --- | --- | --- | --- | --- | --- |
| Nakić, J., Stilin, A., & Tomljenović, L. (2015). The research of interest for the development of sport-based entrepreneurship. *Zbornik Veleučilišta u Rijeci*, *3*(1), 1-11. | Croatia | Quantitative questionnaire | “The use of social issues to create change in the sporting context.” Social entrepreneurship uses sport to encourage solutions to social problems | No | Borne in local context |
| Sunio, V., Laperal, M., & Mateo-Babiano, I. (2020). Social enterprise as catalyst of transformation in the micro-mobility sector. *Transportation Research Part A: Policy and Practice*, *138*, 145-157. | Philippines | Case-study: autoethnography | Community-based social enterprise’ (Cieslik, 2016, Lumpkin et al., 2018. | No | Borne in local context; local language and colloquial preservation; practical application for local context |
| Ebrahimi, A., Doosti, M., Razavi, M. H., & Seifari, M. K. (2019). The cultural strategies for sport entrepreneurship development in Iran. *Sport Science*, *12*(Suppl. 1), 52-55. | Iran | Qualitative: content analysis | Sport-based entrepreneurship: a promising conceptual joint  venture between entrepreneurship and sport (Hemme et al.,  2016), used to meet social needs. | No | Borne in local context |
| Morgado Ramiro de Lima, A., Morgado Ramiro de Lima, G., and Cister, A. (2015). Social Inclusion as Innovation. *Journal of Business and Economics,* 6 (7) pp. 1295 –1298. DOI: 10.15341/jbe(2155-7950)/07.06.2015/006 | Brazil | Case-study | Social Inclusion: André and Abreu (2006, p. 124); as Social Innovation: “refers to innovative activities and services motivated to meet a social need, predominantly disseminated through an organization with social goals.” Regarding André and Abreu (2006, p. 125), | No | Borne in local context |
| Rodrigues, R., Marques, C. S., Esteves, D., Brás, R., Santos, G., Gouveia, A., ... & Marques, V. (2020). Physical activity level as a booster of entrepreneurial intention: a social innovation approach. *International Review on Public and Nonprofit Marketing*, *17*, 121-133. | Portuguese regions | Quantitative: questionnaires ei scale developed by liñán and chen  (2009). | Entrepreneural intention, social innovation | No | N/a |
| Arnold, D., & DeWald, E. (2011). Cycles of empowerment? The bicycle and everyday technology in colonial India and Vietnam. *Comparative Studies in Society and History*, *53*(4), 971-996. | India, indochina | Historical recount | Entrepreneurship; social change; innovation; technological transfer; global diffusion; social life of things | Yes | Local language and colloquial preservation |
| Hayhurst, L. M. (2014). The ‘Girl Effect’and martial arts: Social entrepreneurship and sport, gender and development in Uganda. *Gender, place & culture*, *21*(3), 297-315. | Uganda | Ethnography | Social entrepreneurship, post-feminism | Yes | Reflexivity;  Alternative onto-epistemological logic; local language and colloquial preservation |
| Ardizzi, M., Wilson, B., Hayhurst, L., & Otte, J. (2020). “People still believe a bicycle is for a poor person”: Features of “bicycles for development” organizations in Uganda and perspectives of practitioners. *Sociology of Sport Journal*, *38*(1), 36-49. | Uganda | Critical interpretivist | Post/Colonialism and SDP; International Development; Neoliberalism, and Sport | Yes | Borne in local context; reflexivity; alternative onto-epistemological logic; local language and colloquial preservation;  Practical application or implications for the local context |
| Svensson, P. G., & Seifried, C. S. (2017). Navigating plurality in hybrid organizing: The case of sport for development and peace entrepreneurs. *Journal of Sport Management*, *31*(2), 176-190. | Global | Qualitative: semi-structured interviews | SDP, Battilana  and Lee (2014) introduced the concept of “hybrid  organizing” “as the activities, structures, processes and  meanings by which organizations make sense of and  combine aspects of multiple organizational forms” (p.  398). | Yes | Reflexivity; |
| Hayhurst, L. (2011). *" Governing" the" Girl Effect" Through Sport, Gender and Development? Postcolonial Girlhoods, Constellations of Aid and Global Corporate Social Engagement*. University of Toronto. | Uganda | Post-feminist participatory action research | King (2001) uses the term “global  strategic community relations programs” (GSCR) | Yes | Reflexivity |
| HAO, C., ZOU, X., & BAI, Y. (2020). Social Entrepreneurship of Sports Enterprises Under COVID-19 Epidemic. *Journal of Shenyang Sport University*, *39*(4), 82-86. | China | Comparative case-study | Social entrepreneurship is an important way for enterprises to use commercial means to provide solutions to social problems. | No | Borne in local context |
| Wilson, B., Van Luijk, N., & Boit, M. K. (2015). When celebrity athletes are ‘social movement entrepreneurs’: A study of the role of elite runners in run-for-peace events in post-conflict Kenya in 2008. *International review for the sociology of sport*, *50*(8), 929-957. | Kenya | Qualitative interviews | SDP, social movement theory = ‘resource mobilization  theory’, which is based on the idea that movements are more or less successful  depending on the ability of movement members to access the necessary resources to  support their aims (mccarthy and Zald, 1977) | Yes | Reflexivity; practical application for local context |

Table 1 TABLE OF DATA

**Supplementary Table 1.** Table to display data collected from the integrated review process.

# Table of Conclusions

| **What is the dominant (Global North) discourse on SES** | **What this review adds** |
| --- | --- |
| SES theory privileges the Eurocentric views of entrepreneurship | An alternative (peripheral country) perspective and adds plurality to the concepts and definitions of SES |
| SES is viewed through masculine perspectives of entrepreneurship like perceptions for 'empowerment' and the 'entrepreneur as a sole hero' | Entrepreneurship is viewed in terms of community enterprising |
| SES is often based on colonial tenets | SES is viewed through a decolonial feminist lens (to some degree) |
| SES and social innovation are distinct concepts | Social inclusion in sport is a form of social innovation in sport, which is also defined as social entrepreneurship in sport |
|  | Most authors of SES in a peripheral context are from said context or encultured in the context |
|  | Qualitative methods and exploratory approaches are prevalent in SES research from a (or about) a peripheral country context |

Table 2 TABLE OF CONCLUSIONS

**Supplementary Table 2.** Table to display the conclusions made from data subgroups.

# PRISMA Flow Chart

##
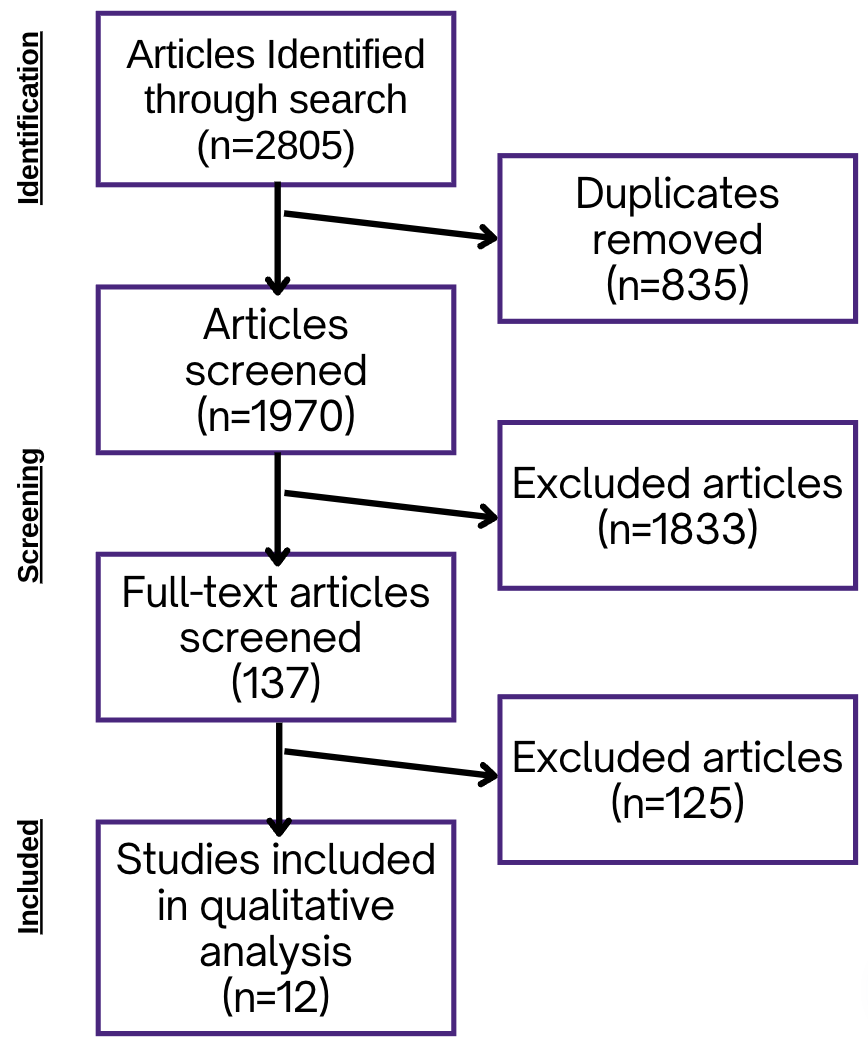


Figure 1 PRISMA FLOW CHART

**Supplementary Figure 1.** PRISMA flow chart of Covidence literature review and data collection process.
